# Psychometric properties and cross‐cultural adaptation of the Japanese version of the Affective Reactivity Index: A community‐based study of adolescent irritability

**DOI:** 10.1002/pcn5.70169

**Published:** 2025-08-04

**Authors:** Makoto Osada, Hiroyuki Mori, Michio Takahashi, Hiroki Shinkawa, Masaki Adachi, Minami Adachi, Takuya Saito, Kazuhiko Nakamura

**Affiliations:** ^1^ Department of Neuropsychiatry, Graduate School of Medicine Hirosaki University Hirosaki Japan; ^2^ Faculty of Humanities Saitama Gakuen University Kawaguchi Japan; ^3^ Smart‐Aging Research Center Tohoku University Sendai Japan; ^4^ Faculty of Education Hirosaki University Hirosaki Japan; ^5^ Department of Psychology Meiji Gakuin University Minato‐Ku Japan; ^6^ Department of Psychiatry of Childhood Puberty, Graduate School of Medicine Hokkaido University Sapporo Japan

**Keywords:** irritability, Affective Reactivity Index, adolescent, psychometric properties, cross‐cultural adaptation

## Abstract

**Aim:**

Self‐reported scales that adequately measure tonic irritability and provide international comparability are lacking in Japan. Therefore, the present study aimed to examine the reliability, validity, and cross‐cultural adaptation of both self‐ and parent‐reported versions of the Japanese version of the Affective Reactivity Index.

**Methods:**

We used 2019 data from the Hirosaki City School Cohort Study, which is a part of the Assessment of Preschool to Adolescence‐Longitudinal Epidemiological Study. To evaluate the construct validity of the Affective Reactivity Index, we examined the association between depressive symptoms and emotional and conduct problems. We applied item response theory to evaluate the psychometric properties of the Affective Reactivity Index and assess various latent traits.

**Results:**

Our findings confirmed the reliability and construct validity of both the self‐ and parent‐reported versions of the Affective Reactivity Index (*α* = 0.86 and 0.82, respectively). The item response theory analysis confirmed the psychometric properties of these measures in a Japanese context.

**Conclusion:**

This study comprehensively examined the reliability, validity, and cross‐cultural adaptation of the Japanese version of the Affective Reactivity Index for both self‐ and parent‐reports. These findings mark a crucial step in developing culturally adapted tools for assessing irritability in Japanese adolescents, contributing to both research and clinical settings.

## INTRODUCTION

Irritability, characterized by easy annoyance and touchiness, accompanied by anger and temper outbursts, is a common symptom of depression in children and adolescents.^1^ It is a prevalent reason for referral to child and adolescent mental health services and plays a crucial role in diagnosing depression and dysthymia in children and adolescents.^2^, ^3^ Notably, children experiencing severe irritability have been identified as having a higher risk of developing anxiety and depressive disorders in the future. Consequently, irritability in children and adolescents is a crucial issue that requires careful attention.^4^

Existing measures often fail to distinguish irritability from related constructs, such as aggression or oppositional behavior.^5^ However, irritability can occur independently of aggressive outbursts^6^ and may also manifest as a symptom of depression in young people.^1^ Thus, accurately distinguishing irritability from related presentations is crucial for proper assessment and intervention. To address this need, the Affective Reactivity Index (ARI) was developed with a specific focus on irritability, deliberately distinguishing it from related constructs, such as aggression or hostility.^7^, ^8^ The ARI offers several advantages over existing measures. First, it employs terms that are easily comprehensible to children and adolescents and are suitable for epidemiological studies because of their concise nature.^6^ Second, recognizing the importance of multi‐informant assessments in child and adolescent mental health, the ARI provides both self‐ and parent‐reported versions.^9^, ^10^ This dual‐perspective approach enhances the comprehensiveness and accuracy of irritability assessments in young populations.

In Japan, irritability is primarily assessed using the Aberrant Behavior Checklist, which emphasizes severe behavioral outbursts, including violent behavior toward oneself, self‐injury, and sudden mood changes for samples with intellectual disabilities.^11^, ^12^ However, this approach fails to capture the full construct of irritability, which encompasses both tonic (persistent angry mood) and phasic (behavioral outbursts) components.^13^, ^14^ Therefore, there is a lack of self‐report scales in Japan that adequately measure tonic irritability and provide international comparability. Also, there are no scales for measuring irritability in general population samples in Japan. Furthermore, Japan's cultural background emphasizes emotional suppression, which differs from Western norms.^15^, ^16^, ^17^ Thus, validating the self‐ and parent‐report versions of the Japanese ARI is essential for assessing irritability and detecting potential depressive symptoms in children and adolescents. Previous studies have validated the ARI in Western and non‐Western contexts, demonstrating strong psychometric properties across different populations.^18^ However, its applicability in Japan, where cultural norms influence emotional expression, remains unexplored.

Therefore, the present study aimed to examine the reliability, validity, and cross‐cultural adaptation of both self‐ and parent‐report (ARI‐P) versions of the Japanese ARI. To evaluate the construct validity of the ARI, we examined the association between depressive symptoms and emotional and conduct problems. Irritability may also manifest as a symptom of depression among youth.^1^ Furthermore, irritability, as measured using the ARI‐P, is moderately associated with emotional and conduct problems.^8^ This study provides a validated measure for assessing irritability in Japanese youth, facilitating cross‐cultural research and improving early detection of mental health concerns in clinical and epidemiological settings.

## METHODS

### Participants

This study used 2019 data from the Hirosaki City School Cohort Study, which is part of the Assessment of Preschool to Adolescence‐Longitudinal Epidemiological (APPLE) Study. The APPLE Study is a comprehensive cohort study investigating mental health in a sample of Japanese children and adolescents.^19^ The school cohort study was conducted every September at all public elementary and junior high schools in Hirosaki City, Japan. In 2019, 11,554 students were eligible to participate in this study. The analysis included fourth‐grade primary school students (9–10 years old) to third‐grade junior high school students (14–15 years old) and their parents/guardians. Of the 7956 student–parent/guardian pairs, 6432 completed the questionnaire (3152 males, 3279 females, and one other; valid response rate: 80.84%; Table 1).

**Table 1:** Descriptive statistics of the school cohort study in Hirosaki City (2019) for ARI and ARI‐P.

Grade	Sample size, *n*	ARI, Mean (SD)	ARI‐P, Mean (SD)
4th	1121	2.14 (3.12)	1.87 (2.54)
5th	1080	1.69 (2.61)	1.62 (2.42)
6th	1106	1.63 (2.70)	1.50 (2.33)
7th	1097	1.44 (2.43)	1.47 (2.24)
8th	1067	1.33 (2.29)	1.41 (2.26)
9th	953	1.35 (2.41)	1.32 (2.12)
Total	6432	1.42 (2.31)	1.40 (2.06)
Male	3152	1.70 (2.71)	1.54 (2.37)
Female	3279	1.52 (2.54)	1.54 (2.29)

Abbreviations: ARI, Japanese version of the Affective Reactivity Index self‐report; ARI‐P, Japanese version of the Affective Reactivity Index parent‐report; SD, standard deviation.

### Ethical approval

This study was approved by the Ethics Committee of Hirosaki University Graduate School of Medicine (IRB# 2015–055). Prior to data collection, the researchers distributed a document explaining the purpose of the study and the voluntary nature of participation to the students and their parents/guardians via classroom teachers.

### Measures

#### Affective Reactivity Index

The ARI was developed to measure irritability in both self‐report (ARI) and ARI‐P versions, using identical items for both versions. Respondents are required to rate irritability over the last 6 months.^8^ The scale comprises six items assessing irritability‐related feelings and behaviors, plus one item evaluating impairment because of irritability (“overall, irritability causes him/her [or ‘me’ in self‐report] problems”). Each item is rated on a three‐point Likert scale: 0 (*not true*), 1 (*somewhat true*), and 2 (*certainly true*). The total score is calculated by summing the responses to the first six items, resulting in a possible score range of 0–12 points. Higher scores indicate greater levels of irritability. Tseng et al.^20^ reported excellent test–retest reliability over a 4‐week period for both the ARI (item characteristic curve [ICC] = 0.88, *p* < 0.001) and ARI‐P (ICC = 0.90, *p* < 0.001) versions. The ARI has also been used in school‐aged children (9 years and older).^18^, ^20^ The Japanese versions of ARI and ARI‐P are not publicly available. However, one of the authors, T.S., obtained permission from the original author, Dr. Argyris Stringaris, to use an authorized Japanese translation of the ARI and ARI‐P. We performed a forward‐translation and back‐translation of the ARI and ARI‐P, and the back‐translated version was carefully examined by a bilingual research psychologist, who confirmed the semantic and conceptual equivalence between the original and the Japanese versions. Once forward‐translation and back‐translation had been submitted to Dr. Argyris Stringaris, any discrepancies of translation were reviewed by Dr. Gail Tripp and Emi Furukawa, PhD from the Okinawa Institute of Science and Technology Graduate University. Thereafter, Dr. Argyris Stringaris made final adjustments and authorized the official version.

#### Strengths and Difficulties Questionnaire

We employed the Japanese version of the Strengths and Difficulties Questionnaire (SDQ) for the parent‐reports. The SDQ is a well‐established measure with evidence of internal consistency and validity.^21^, ^22^ It consists of 25 items rated on a 3‐point Likert scale (*not true*, *somewhat true*, and *certainly true*). The items are grouped into five subscales of five items each: Emotional Problems, Conduct Problems, Hyperactivity, Peer Problems, and Prosocial Behavior. The Japanese version of the SDQ demonstrates adequate reliability and validity.^23^ Higher scores on the four problem subscales and the total difficulties score indicate greater levels of psychological difficulties. The Prosocial subscale is scored in the opposite direction, with higher scores indicating better prosocial behavior.

#### Patient Health Questionnaire for Adolescents

The Patient Health Questionnaire for Adolescents (PHQ‐A) is a self‐report questionnaire adapted from the adult version of the Patient Health Questionnaire‐9, which is one of the most widely used screening tools for major depression worldwide.^24^ Its nine components align with the “A” diagnostic criteria for major depression, and its psychometric properties have been confirmed for Japanese adolescents.^25^ Each item is rated on a four‐point Likert scale: 0 (*not at all*), 1 (*several days*), 2 (*more than half the days*), and 3 (*nearly every day*). The total score is calculated by summing the responses to the nine items, resulting in a possible score range of 0–27 points. Higher scores indicate more severe depressive symptoms.

### Analytic strategy

First, Cronbach's alpha coefficients were calculated for both the ARI and ARI‐P to examine the internal consistency of the Japanese version. Confirmatory factor analysis (CFA) was performed on both the ARI and ARI‐P to examine the factor structure of the Japanese version. Model fit was evaluated using the following indices: comparative fit index (CFI), with values > 0.90 indicating adequate fit and values between 0.95 and 0.99 indicating excellent fit^26^; the root‐mean‐square error of approximation (RMSEA), with values ≤ 0.05 indicating close fit and values between 0.05 and 0.10 indicating adequate fit^27^; and standardized root‐mean‐square residual (SRMR), with the cutoff value under 0.08.^28^

Second, we conducted correlation analyses to examine the relationships between the total scores of the ARI and ARI‐P, ARI and PHQ‐A scores, and ARI‐P and SDQ subscale scores. Multiple regression analyses were conducted to examine the construct validity of the Japanese versions of the ARI‐P. As in a previous study,^8^ analyses were conducted as follows: the first analysis with emotional problems as the dependent variable, where the other subscales of the SDQ were entered as control variables and ARI‐P was entered as the independent variable; and the second analysis with conduct problems as the dependent variable, where the other subscales of the SDQ were entered as control variables, and ARI‐P was entered as the independent variable. Since the self‐report SDQ was not included in this study, correlation analysis was performed with PHQ‐A to validate the validity of the ARI.

Finally, descriptive statistics of the means and standard deviations (SDs) were calculated for the ARI and ARI‐P items and total scores. Item response theory (IRT) was applied to both the ARI and ARI‐P to examine the basic psychometric properties and a range of latent traits using a graded response model.^29^ We examined the ICCs and test information functions (TIFs) for both ARI and ARI‐P. ICCs were analyzed to examine the psychometric properties of each item in the Japanese versions of the ARI and ARI‐P. These curves provide a visual representation of how each item functions across different levels of latent traits. The Japanese and other country versions of the TIFs were compared to evaluate whether there was equivalence in measurement precision between them.^30^, ^31^ Discrimination and difficulty parameters were used to generate ICCs, which were aggregated across the items to generate TIFs. This function indicates the spread across *θ*, at which a measure provides reliable information. The discrimination (*a* > 0.50) and difficulty (−4.0 < *b* < 4.0) parameters were evaluated based on the criteria suggested by Roznowski.^32^

Missing values were imputed using the maximum likelihood method. Analyses were performed using SPSS (Ver. 28), AMOS (Ver. 28), and HAD.^33^ Statistical significance was set at *p* < 0.05.

## RESULTS

### Cronbach's alpha coefficients and CFA

Internal consistency was adequate for both the Japanese version of the ARI (*α* = 0.86) and the ARI‐P (*α* = 0.82). Table 2 presents item analyses for the Japanese versions of the ARI and ARI‐P.

**Table 2:** Item analyses for Japanese versions of the ARI and ARI‐P.

	Items	*M*	SD	Factor loadings		Items	*M*	*SD*	Factor loadings
ARI	ARI Total	1.42	2.31		ARI‐P	ARI‐P Total	1.40	2.06	
Stringaris et al.^8^		(3.32)	(NA)		Stringaris et al.^8^		(3.34)	(NA)	
α = 0.85	ARI‐1	0.47	0.65	0.61	α = 0.82	ARI‐P‐1	0.48	0.62	0.65
(α = 0.90)		(0.74)	(0.69)	(0.90)	(α = 0.89)		(0.84)	(0.77)	(0.68)
	ARI‐2	0.26	0.52	0.73		ARI‐P‐2	0.27	0.53	0.77
		(0.62)	(0.75)	(0.96)			(0.69)	(0.80)	(0.97)
	ARI‐3	0.19	0.48	0.71		ARI‐P‐3	0.14	0.39	0.55
		(0.52)	(0.71)	(0.78)			(0.49)	(0.72)	(0.81)
	ARI‐4	0.15	0.42	0.74		ARI‐P‐4	0.09	0.31	0.58
		(0.30)	(0.58)	(0.88)			(0.16)	(0.40)	(0.82)
	ARI‐5	0.23	0.51	0.79		ARI‐P‐5	0.28	0.51	0.73
		(0.46)	(0.71)	(0.94)			(0.51)	(0.72)	(0.97)
	ARI‐6	0.14	0.41	0.75		ARI‐P‐6	0.14	0.40	0.76
		(0.68)	(0.79)	(0.93)			(0.65)	(0.72)	(0.97)
	ARI‐7	0.18	0.47	—		ARI‐P‐7	0.14	0.40	—

*Note*: ARI‐7 and ARI‐P‐7 don't include total score of the ARI and ARI‐P.

Abbreviations: ARI, Japanese version of the Affective Reactivity Index self‐report; ARI‐P, Japanese version of the Affective Reactivity Index parent‐report; M, mean; NA, not applicable; SD, standard deviation.

CFA was conducted on both Japanese versions. Factor loadings for the Japanese ARI (0.61–0.79) were lower than those previously reported,^8^ but a one‐factor model demonstrated excellent fit according to CFI and SRMR, and adequate fit based on RMSEA (χ^2^(9) = 627.18, *p* < 0.05, SRMR = 0.03, CFI = 0.96, RMSEA = 0.10, 90% confidence interval [CI]: 0.10–0.11), consistent with recommended thresholds.^26^, ^27^, ^28^ Similarly, the factor loadings for the Japanese ARI‐P were lower than those of the original version^8^ and yielded a one‐factor model in *χ* ^*2*^(9) = 1333.90 (*p* < 0.01). While SRMR (0.05) and CFI (0.90) indicated an acceptable model fit,^26^, ^28^ RMSEA (0.15, 90% CI: [0.15–0.16]) exceeded the recommended threshold by Browne and Cudeck.^27^

### Correlation and multiple regression analyses for confirming construct validity

Correlation analysis revealed that the association between the total scores of the Japanese ARI and ARI‐P was weak (*r* = 0.26, *p* < 0.001). In contrast, a moderate correlation was observed between the ARI and PHQ‐A (*r* = 0.57, *p* < 0.001).

Table 3 presents the correlations between the ARI‐P and SDQ subscales. The ARI‐P was associated with prosocial behavior, hyperactivity, emotional problems, and peer problems. a strong correlation was found with conduct problems.

**Table 3:** Correlation coefficient (*r*) between the ARI‐P and SDQ subscales.

Variables	ARI‐P (*r*)	
Emotional Problems	0.34	[***]
Conduct Problems	0.62	[***]
Hyperactivity	0.33	[***]
Peer Problems	0.27	[***]
Prosocial Behavior	−0.17	[***]

*Note*: Emotional Problems, Conduct Problems, Hyperactivity, Peer Problems, and Prosocial Behavior are SDQ subscales.

Abbreviations: ARI‐P, Japanese version of Affective Reactivity Index parent‐report; SDQ, Strengths and Difficulties Questionnaire.

*** *p* = 0.001.

The results of the multiple regression analysis (forced entry method) with emotional and conduct problems as independent variables and other SDQ subscales and ARI‐P as independent variables are presented in Table 4. The ARI‐P showed a significant positive association with emotional problems. Additionally, it showed a moderately positive association with conduct problems. These results indicate that parent‐reported irritability, as measured by the ARI‐P, is significantly associated with both emotional and conduct problems.

**Table 4:** Results of multiple regression analysis (ARI‐P).

Variables	Emotional problems (*β*)		Conduct problems (*β*)	
ARI‐P	0.25	[***]	0.50	[***]
Emotional Problems	—	—	−0.02	[*]
Conduct Problems	−0.04	[*]	—	—
Hyperactivity	0.18	[***]	0.31	[***]
Peer Problems	0.29	[***]	0.00	NS
Prosocial Behavior	0.14	[***]	−0.15	[***]
*Adj. R*²	0.23	[***]	0.50	[***]

*Note*: Emotional Problems, Conduct Problems, Hyperactivity, Peer Problems, and Prosocial Behavior are Strengths and Difficulties Questionnaire subscales.

Abbreviations: ARI‐P, Japanese version of Affective Reactivity Index parent‐report; NS, not significant.

* *p* < 0.05.

*** *p* < 0.001.

### Descriptive statistics and IRT for the graded response model

**Figure 1:**
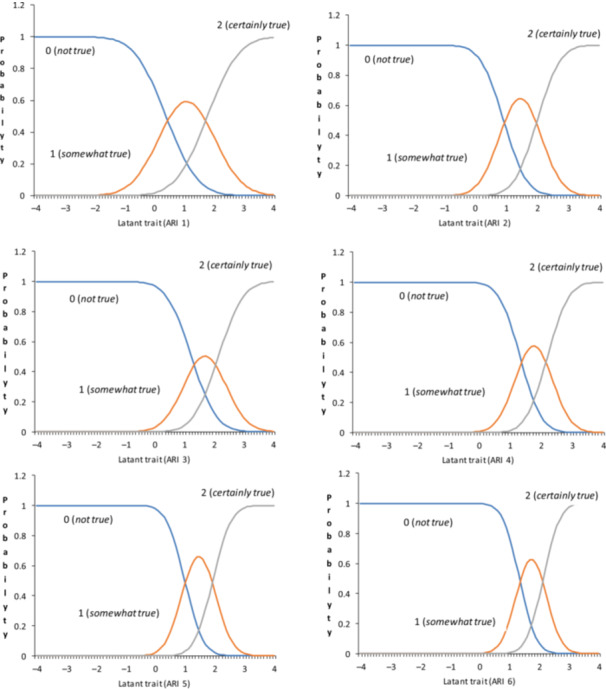
Item characteristics curves of the Japanese version of the Affective Reactivity Index self‐report (ARI).

**Figure 2:**
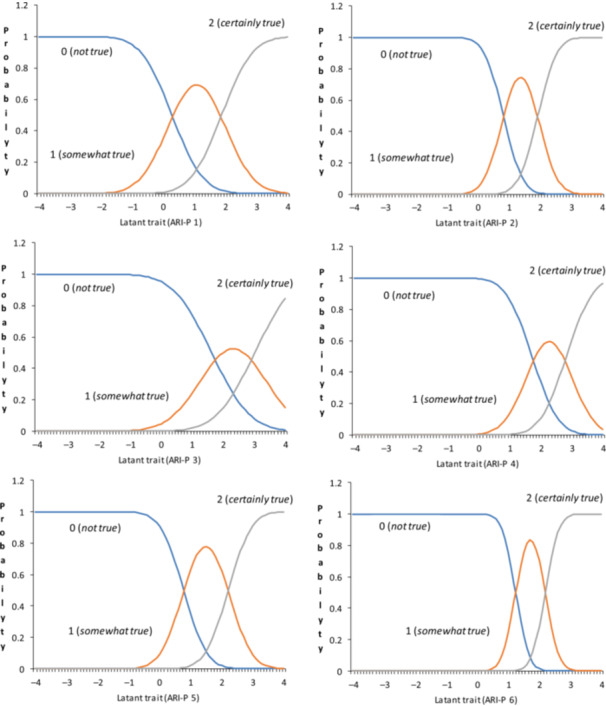
Item characteristics curves of the Japanese version of the Affective Reactivity Index parent‐report (ARI‐P).

Descriptive statistics were calculated for both the Japanese ARI and ARI‐P (Table 5). The mean and standard deviation (SD) for each item in the Japanese ARI and ARI‐P were lower than those in the original UK ARI and ARI‐P samples.^8^ Next, an IRT analysis using the graded response model was conducted for ARI and ARI‐P. The discrimination and difficulty parameters for both ARI and ARI‐P are shown in Table 5. For the ARI, the discrimination parameters ranged from 1.19 to 2.24, with Item 6 showing the highest discrimination (2.24) and Item A1 the lowest (1.19). The difficulty estimates for the first threshold (*b1*) ranged from 0.39 to 1.34, whereas those for the second threshold (*b2*) ranged from 1.78 to 2.21. Regarding difficulty parameters, Item 1 demonstrated the lowest threshold estimates (*b1* = 0.39, *b2* = 1.78), whereas Items 3, 4, and 6 showed the highest estimates (*b2* = 2.14, 2.21, and 2.14, respectively). For ARI‐P, the discrimination parameters ranged from 1.03 to 2.91. Item 6 of the ARI‐P similarly displayed the highest discrimination value (*α* = 2.91). In terms of difficulty, the parameters ranged from 0.28 to 1.72 for b1 and from 1.87 to 3.01 for b2. Item 1 had the lowest thresholds (*b1* = 0.28, *b2* = 1.87), whereas Item 3 exhibited the highest thresholds (*b1* = 1.62, *b2* = 3.01). ICCs for both the ARI and ARI‐P indicated robust discrimination across all items (*α* > 1.0 for all items) and were particularly suited for assessing moderate‐to‐high irritability levels, as evidenced by the predominantly positive difficulty parameters (Figures 1 and 2).

**Table 5:** Item parameters for the Japanese versions of the ARI and ARI‐P.

		Discrimination	Difficulty	
Items		*a*	*b1*	*b2*
ARI‐1	Easily annoyed by others	1.19	0.39	1.78
ARI‐2	Often lose temper	1.71	0.92	2.01
ARI‐3	Stay angry for a long time	1.50	1.23	2.14
ARI‐4	Angry most of the time	1.84	1.34	2.21
ARI‐5	Get angry frequently	2.03	0.99	1.93
ARI‐6	Lose temper easily	2.24	1.34	2.14
ARI‐P‐1	Easily annoyed by others	1.27	0.28	1.87
ARI‐P‐2	Often lose temper	2.05	0.82	1.93
ARI‐P‐3	Stay angry for a long time	1.03	1.62	3.01
ARI‐P‐4	Angry most of the time	1.51	1.72	2.82
ARI‐P‐5	Get angry frequently	1.70	0.77	2.21
ARI‐P‐6	Lose temper easily	2.91	1.24	2.19

*Note*: ARI, Japanese version of Affective Reactivity Index self‐report; ARI‐P, Japanese version of Affective Reactivity Index parent‐report.

**Figure 3:**
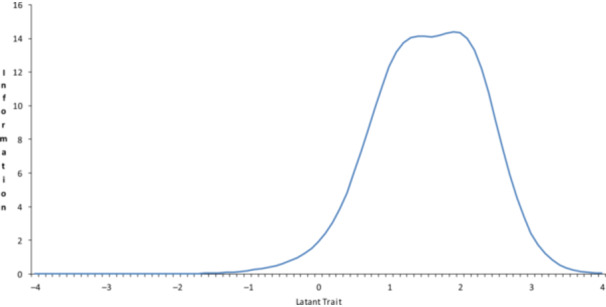
Test information functions of the Japanese version of the Affective Reactivity Index self‐report.

**Figure 4:**
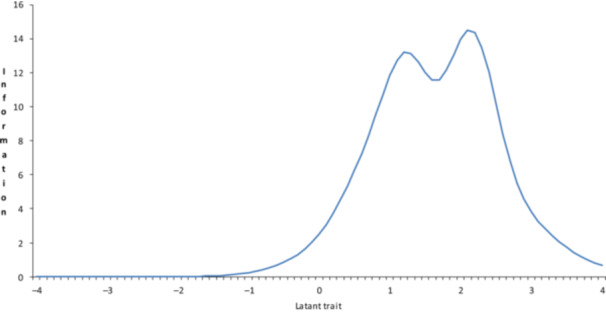
Test information functions of the Japanese version of the Affective Reactivity Index parent‐report.

The test information curve for both ARI and ARI‐P exhibited bell‐shaped distributions that were slightly skewed to the right of the latent trait continuum. The ARI demonstrated optimal measurement precision in the range of *θ* = 0 to +2, with peak information content in the *θ* = +1 to +2 range (Figure 3). The ARI‐P showed high information content in the θ range of 0 to +2, with two distinct peaks observed at approximately *θ* = +1 and +2 (Figure 4).

## DISCUSSION

This study aimed to examine the reliability, validity, and cross‐cultural adaptation of the Japanese versions of the ARI and ARI‐P. Our findings support the reliability and construct validity of these two indices. While our analysis confirmed the original one‐factor structure of the ARI, the Japanese version of the ARI‐P exhibited suboptimal model fit for certain indicators. IRT analysis validated the psychometric properties of these measures in the Japanese context.

The Japanese versions of the ARI and ARI‐P demonstrated adequate internal consistency comparable to that of the original English version.^8^ This suggests that both scales are reliable measures of irritability among Japanese children and adolescents.

CFA supported the one‐factor structure of the Japanese version of the ARI, which was consistent with the original version.^8^ For the ARI‐P, based on the criteria proposed by Browne and Cudeck,^27^ the CFI and SRMR values were within the acceptable range. Although the RMSEA values exceeded the recommended threshold, model fit indices should be interpreted with flexibility.^34^ In addition, considering that the original version by Stringaris et al.^8^ demonstrated a one‐factor structure, the adoption of a one‐factor structure in this study is justified both theoretically and empirically. In the American early adolescent sample^35^ and the Australian adolescent sample,^18^ the CFA for the ARI‐P showed an adequate model fit in the single‐factor structure model. These discrepancies in the results could be attributed to cultural differences. In Japanese culture, emotional restraint is often valued, which differs from Western norms.^15^, ^16^, ^17^ Nevertheless, it is important to acknowledge that there is room for improvement in the model fit. Cultural differences may have influenced the increase in the RMSEA of ARI‐P. Future research should focus on exploring alternative factor structures that account for cultural norms related to emotional expression, the cultural adaptation of the items and their validation across diverse regions in Japan and other countries to enhance the scale's psychometric properties and cultural relevance.

This study provides evidence for the construct validity of the Japanese versions of the ARI and ARI‐P, while also highlighting some cultural considerations. A significant, strong positive correlation was observed between the ARI and the PHQ‐A. This finding aligns with those of previous studies^1^, ^18^ and supports the construct validity of the Japanese version of the ARI. The correlation between the ARI and ARI‐P (*r* = 0.26, *p* < 0.001) was weaker than that in the original study; however, it was within the range typically observed in parent–child agreement on behavioral measures.^36^, ^37^ This level of agreement is consistent with findings from other community samples, including those reported by Dougherty et al.,^35^ suggesting that while parent and child reports provide related information, they also offer unique perspectives on the child's irritability.

Multiple regression analyses revealed significant associations between irritability, as measured by the ARI‐P, and emotional and conduct problems. This was partially different from the original finding that the ARI‐P did not predict conduct problems.^8^ However, our results align with more recent research on the associations between irritability and conduct problems, such as the diagnosis of disruptive mood dysregulation disorder (DMDD)^38^ and severe mood dysregulation.^3^ This finding emphasizes the complex relationship between irritability and externalizing behaviors and highlights the importance of considering the cultural context in interpreting these relationships.^39^

Descriptive statistics and IRT analysis revealed that both the Japanese ARI and ARI‐P demonstrated good psychometric properties. The total scores for both versions were lower than those reported for the original UK sample.^8^, ^40^ These findings align closely with data from Australia^18^ and another Japanese sample,^41^ suggesting a potential cultural influence on irritability reporting. The lower scores in the Japanese samples may reflect a lower prevalence of mood disorders in Japan than in Western countries.^42^, ^43^ This discrepancy may reflect cultural differences in the expression of mental health symptoms, stigma surrounding mental disorders, or the potential inadequacy of Western diagnostic tools in the Japanese context.^44^

The discrimination parameters for all items in the ARI and ARI‐P exceeded 1.0, indicating their effectiveness in distinguishing individuals with different levels of irritability.^31^ The range of threshold parameters suggests that both versions are particularly effective in assessing moderate‐to‐high levels of irritability. Item 6 (“Lose temper easily”) demonstrated the highest discrimination parameter in both Japanese versions of the ARI (self‐report: *α* = 2.24; parent‐report: *α* = 2.91), consistent with findings from the Brazilian^45^ and US^35^ samples.

The difficulty parameters revealed that Items 3, 4, and 6 had the highest thresholds (*b2* = 2.14, 2.21, and 2.14, respectively) for the ARI, indicating that respondents who select higher response categories for these items are likely to experience more intense irritability. This finding is partly in line with those of previous research but also reveals some cultural differences. In the US sample,^35^ Item 3 (“Gets angry frequently”) also demonstrated high difficulty, suggesting a cross‐cultural consistency in the perception of frequent anger as indicative of more severe irritability. However, the high difficulty of Item 4 (“Angry most of the time”) in our Japanese sample, while consistent with the Brazilian sample,^45^ differs from the US findings. This discrepancy may reflect cultural variations in the interpretation and expression of prolonged anger. For the ARI‐P, Item 3 had the highest threshold (*b1* = 1.62, *b2* = 3.01), and parents endorsing higher categories of Item 3 reported more severe levels of irritability. This finding is consistent with that of a previous study.^35^ This cross‐cultural consistency suggests that losing one's temper is a robust and persistent indicator of irritability, thus supporting the construct validity of the ARI. The item's high discrimination across cultures corresponds with the diagnostic criteria for irritability‐related disorders, such as DMDD,^38^ supporting the clinical utility of the ARI in diverse cultural settings. However, discrepancies in the difficulty of the ARI between the previous and present studies might reflect cultural differences in the expression and perception of irritability, particularly in the Japanese culture, where emotional restraint is often valued.^15^, ^16^, ^17^ These findings underscore the importance of considering both universal and culture‐specific aspects of irritability in cross‐cultural research and clinical practice.^46^

In this study, the item parameters of the Japanese versions of the ARI and ARI‐P exhibited consistency in the ICCs (Figures 1 and 2), indicating equivalent item functioning across both versions. This finding supports both versions that can measure the same components consistently.

In addition, the test information curve for both versions supports these findings (Figures 3 and 4), demonstrating optimal measurement precision for moderate‐to‐high irritability levels (*θ* = 0 to +2). This property is crucial for clinical assessment and research applications, as it allows for the accurate identification of clinically significant irritability levels. However, the reduced discriminative ability at lower irritability levels in general population samples suggests that these measures may be more suitable for clinical populations or for identifying youth at risk of psychopathology. Notably, the self‐reported ARI showed a more continuous measurement profile than the parent‐reported version. This difference might reflect variations in how youth perceive and report their own irritability compared to parental observations, a phenomenon observed in other areas of child and adolescent assessment.^10^ These results are consistent with findings in US and Brazilian samples^35^, ^45^ and support the potential utility of the scale in identifying clinically significant irritability. In short, the Japanese versions of both the ARI and ARI‐P are consistent with the objectives of the original scale, which was designed as a brief measure of irritability for clinical research.^8^ This study suggests that the Japanese versions of the ARI and ARI‐P are appropriate for identifying psychiatric irritability in Japan.

### Strengths and limitations

This study has several strengths, as it provides a comprehensive examination of the reliability, validity, and cross‐cultural adaptation of the Japanese versions of the ARI and ARI‐P. Both the ARI and ARI‐P demonstrated adequate internal consistency (*α* = 0.86 and 0.82, respectively), comparable to that of the original version. IRT analysis provided in‐depth insights into the measurement properties of the scales. Additionally, this study used data from 6432 participants from a substantial community sample. Large‐scale studies enhance the robustness of reliability and validity estimates and improve the precision of factor analysis.^47^, ^48^ Finally, by confirming the equivalent item functioning of the ARI and ARI‐P, this study contributes to a multifaceted understanding of irritability assessment and a more comprehensive evaluation of the construct of irritability in Japanese adolescents.

Despite these strengths, this study had some limitations. First, although the community‐based sample included **i**ndividuals with elevated scores on the ARI, ARI‐P, SDQ, and PHQ‐A who may meet the clinical criteria, the dataset lacked formal diagnostic information to identify clinical cases. This limits the generalizability of findings to clinical settings. Despite the large community sample, this study was limited to a single region. Further investigation is required to examine the reliability and validity of the ARI and ARI‐P in Japanese clinical samples and in other regions. Second, the results indicated strong precision at moderate to high irritability levels, which is well suited for clinical identification. However, precision was lower for samples with low irritability, reducing sensitivity for population‐based screening. This highlights the need for additional items to capture individual differences in irritability among the general population. Third, although an IRT analysis was conducted, a differential item functioning (DIF) analysis, which directly compares data from other countries and other regions of Japan, was not conducted. Future research should employ DIF analysis to test cross‐national item equivalence and provide robust evidence of cross‐cultural differences in item functioning. Finally, although this study employed a cross‐sectional design, the APPLE Study collected longitudinal data. Future research should verify whether ARI and ARI‐P predict subsequent depression.

## CONCLUSION

This study provides evidence for the reliability, validity, and cross‐cultural adaptation of the Japanese versions of the ARI and ARI‐P in a large community sample of Japanese adolescents. Both measures demonstrated adequate psychometric properties, with the ARI and ARI‐P maintaining the original one‐factor structure. IRT analyses revealed that both versions effectively assessed moderate‐to‐high irritability levels, aligning with their intended clinical use. These findings represent a significant step toward providing culturally adapted tools for assessing irritability in Japanese adolescents, contributing to both clinical practice and cross‐cultural research on child and adolescent mental health.

## AUTHOR CONTRIBUTIONS

Makoto Osada and Hiroyuki Mori conceived and designed the study. Hiroyuki Mori, Michio Takahashi, Hiroki Shinkawa, Masaki Adachi, Takuya Saito, and Kazuhiko Nakamura collected the data. Data analysis was performed by Makoto Osada and Minami Adachi. The figures were drafted by Makoto Osada and Hiroki Shinkawa. Makoto Osada and Hiroyuki Mori wrote the first draft of the manuscript, and all the authors commented on subsequent versions of the manuscript. All authors have read and approved the final manuscript.

## CONFLICT OF INTEREST STATEMENT

The authors declare no conflicts of interest.

## ETHICS APPROVAL STATEMENT

Informed consent was obtained from the patients' legal guardians. Informed consent was obtained from all children participating in the study. This study was approved by the Medical Ethics Committee of Hirosaki University Graduate School of Medicine (IRB# 2015–055).

## PATIENT CONSENT STATEMENT

Consent for publication was obtained from all participants.

## CLINICAL TRIAL REGISTRATION

This study did not require registration as it was not a clinical trial.

## Data Availability

The data, materials, and analytical codes necessary to reproduce the analyses presented herein are not publicly accessible.
